# Building the First Statewide Quality Improvement Collaborative, the CPQCC: A Historic Perspective

**DOI:** 10.3390/children7100177

**Published:** 2020-10-12

**Authors:** Jeffrey B. Gould

**Affiliations:** 1 Department of Pediatrics, School of Medicine, Stanford University, Stanford, CA 94305, USA; jbgould@stanford.edu; 2 California Perinatal Quality Care Collaborative, Stanford, CA 94305, USA

**Keywords:** collaborative quality improvement, perinatal healthcare, neonatology

## Abstract

The California Perinatal Quality Improvement Collaborative (CPQCC), founded in 1997, was the country’s first statewide perinatal quality improvement collaborative. Our goal was to improve the quality and outcomes of perinatal healthcare in California by developing a collaborative network of public and private obstetric and neonatal providers, insurers, public health professionals, and business groups to support a system for benchmarking and performance improvement activities for perinatal care. In this presentation, we describe how viewing the CPQCC as a complex value-driven organization, committed to identifying and addressing the needs of both its stakeholder partners and neonatal intensive care unit (NICU) members, has shaped the course of its development.

## 1. Introduction

An essential factor in the success of any innovative endeavor is to have a clear picture of what you want to achieve, its potential benefits, and even more importantly, the identification of factors that may block innovation, and potential strategies to overcome them. When we first entertained the possibility of California as the site to create the country’s first statewide perinatal quality improvement collaborative, we were told that especially in California, it would be impossible. In the late 1990s, California was known for the competitive fragmentation of the perinatal provider community. Deregionalization had led to competition and distrust between the traditional academic centers and the emerging community-based neonatal intensive care units. Within community providers, there was competition between emerging large-scale multisite provider groups and the more traditional hospital-based neonatal programs. Geographically, there was even open competition between academic referral centers based in northern and southern California.

Engaging the state health systems as essential partners proved to be another challenge. Although it was imperative to recruit California Maternal and Child Health, California Children’s Services, California Vital Records, and Office of State Health Planning and Development (OSHPD) as working partners, these groups tended to exist within independent silos and initially viewed the creation of a statewide, data-driven, provider-based quality improvement organization as trespassing into their specific territories. However, within perinatal medicine, a call for health provider accountability was also emerging both nationally and locally. California’s Pacific Business Group on Health, an essential potential partner representing the interests of payers and by extension, the healthcare of their families, supported the idea of building an organization to assess the quality of care. However, this group’s motivation was consumer advocacy rather than quality improvement. They were gathering, assessing, and report carding their own outcomes data without provider input and were viewed almost as an enemy by the provider community—an enemy whose goal was not to promote quality improvement but to bring about the elimination of low performing institutions. Given the many disparate interests that existed in the mid-1970s, the creation of a statewide California perinatal collaborative built upon the essential partnership of perinatal providers, state health organizations, and consumer advocates seemed all but impossible.

There was, however, a series of events that suggested the possibility of developing a statewide collaborative. Dr. David Stevenson and colleagues had recently developed the California Association of Neonatologists (CAN). Dr. Stevenson felt that, to strengthen CAN’s foundation, it would be important to involve its members in an important and far reaching project. In a conversation with David, I proposed that developing a quality improvement collaborative to serve as its action arm represented an important enterprise for this new organization. He agreed and became instrumental as a cofounder of the California Perinatal Quality Care Collaborative (CPQCC). Another essential partner, the California Maternal, Child, and Adolescent Health (MCAH) branch, was charged with understanding how well California was meeting the needs of pregnant mothers and their newborns.

In the early 1970s, California MCAH developed and published a risk-adjusted neonatal mortality report for every delivery facility in California. The approach created by Dr. Ronald Williams at the University of California, Santa Cruz utilized the paradigm that outcome was a function of risk, care, and chance. After adjusting for differences in case mix and taking chance into account, one could develop an estimate of risk-adjusted neonatal mortality for each California delivery hospital that could be benchmarked against all California facilities. Although well-intentioned, the report was highly technical in its format and its public release was controversial. This led to its being abandoned due to unfavorable acceptance by the provider community. However, by the mid-1990s, in large part due to the publications of the risk-adjusted variation on surgery in New England by Wennberg and Gittelson [1] and outcome variation in chronic lung disease across eight neonatal intensive care units (NICUs) attributed to variation in practice effectiveness by Avery [2], the traditional notion that quality could be assured on the basis of institutional reputation was rapidly being replaced by an emerging need to assess the quality of care based on timely, case mix-adjusted outcome data. Responding to this emerging need, several California hospital systems convinced MCAH to reinitiate these reports. Working in the Maternal Child Health program at the University of California, Berkeley School of Public Health, I had developed for California MCAH one of the country’s first perinatal geographic information systems that combined sociodemographic US census data with data from state birth and death certificate records to profile perinatal risks and outcomes for each zip code in California [3]. Because of the success of this utility in identifying hot spots for adolescent pregnancy intervention [4] as well as other indicators of perinatal need, I was asked to develop and recreate William’s yearly risk-adjusted rates of neonatal mortality for each of California’s ~280 delivery hospitals. Because neonatal mortality is an uncommon event, I felt that collecting data on neonatal morbidity would provide a more sensitive measure of the quality of care and was able to explore the feasibility and approach to risk-adjusted neonatal morbidity analysis as part of the work scope. The big stumbling block in developing this approach was how to obtain morbidity data to perform the analysis.

Dovetailing California’s need for outcomes assessment and CAN’s need for a significant project, we proposed the development of a statewide collaboration of neonatal intensive care and obstetric care providers to both assess and improve perinatal morbidity. We were able to enlist Dr. Rugmini Shah, Director of California’s MCAH, as a cofounding partner. Dr. Shah put us in contact with Dr. Maridee Gregory, the director of California Children’s Services (CCS). CCS paid for the majority of neonatal intensive care in California and had the responsibility to assure that this care was of high quality. In order to do this, they had created a multipage report form that each NICU was required to submit each year. Compliance was extremely poor and even when reports were turned in, the workload at the state did not allow for a careful analysis of these reports. The possibility of an organization that would collect outcomes data and provide risk-adjusted benchmarked estimates of quality care brought CCS on board. The leadership of MCAH and CCS helped to enlist state vital statistics and the OSHPD as members of our executive leadership committee.

At this point, we had enough enthusiastic support for a collaborative to collect risk-adjusted perinatal morbidity data. The remaining challenge was how to build a statewide perinatal database. Once again, serendipity came to the front. The Vermont Oxford Network (VON) was established in 1989 with the primary goal of conducting volunteer-based randomized clinical trials in the NICU following the model developed at Oxford in England. Their first step was to build a multi-institutional database. I was fortunate to be involved as a consultant with Drs. Jerold Lucey and Jeffrey Horbar at VON’s inception and had followed their expansion to NICUs across the US. Although the development of VON had been largely funded by California’s Lucile Packard Foundation, the number of VON NICUs in California in 1996 was only 12. In discussions with Drs. Horbar and Lucey, we explored the possibility of developing a statewide expansion of the VON database as the backbone for the development of the California Perinatal Quality Care Collaborative (CPQCC). Our goal was to focus on quality improvement rather than randomized clinical trials.

In 1997, we applied to the Packard Foundation and were able to put together funding from Packard, state MCAH, and CCS to establish the CPQCC. The plan was to develop the CPQCC as the action arm of CAN with the goal of developing a collaborative *network* of public and private obstetric and neonatal providers, insurers, public health professionals, and business groups to support a system for benchmarking and performance improvement activities for perinatal care. It is important to note that the purpose of the collaborative was not the passive documenting and reporting of outcomes. Our mission was to collect the data needed to inform activities designed to improve perinatal outcomes for all of California. We were fortunate to recruit Dr. David Wirtschafter, one of neonatology’s pioneer quality improvement advocates who stated our task as (1) collecting high-quality reliable data, (2) transforming the data into information by the development of fair risk adjustment and timely reports that inform and organize work, and most importantly, (3) to promote action by supporting perinatal providers in their work of improving perinatal care and outcomes. In addition, we also had an organizational philosophy: (1) That quality improvement was an essential part of perinatal practice, (2) that the collaborative should be bottom-up and provider-driven, and (3) that all aspects of the collaborative should be value-driven. With these as our founding principles and Stanford University School of Medicine agreeing to serve as our administrative intermediary, the CPQCC was launched.

## 2. Ideating the Creation of a Complex Organization

At this point, we had all of the foundational pieces in place: overall objective, initial funding, administrative home, database platform—the big problem was getting our potential partners to work together as an effective executive committee. That is, how do we get these essential partners not only on board but working together to create an enterprise that had both benefits with respect to their strategic mission but also could potentially threaten their autonomy? Although California’s perinatal scene was often described as a hotbed of rivals, the one unifying factor was that all of the potential partners were strongly committed to the goal of improving the health and outcomes of all California mothers and their newborn infants. It is important to note that, in creating the CPQCC’s executive committee, we did not want to recruit members as passive stakeholders but as working partners. The overwhelming challenge at this point was how to get them to work together.

**Figure 1:**
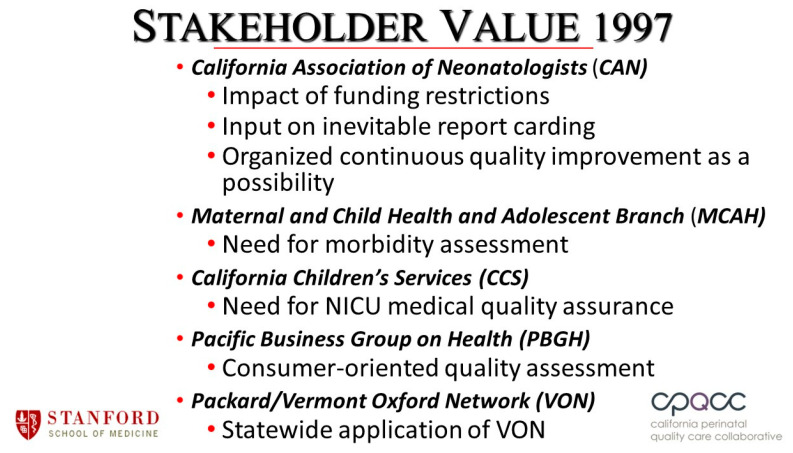
Founding partners and stakeholders involved in the launch of the California Perinatal Quality Improvement Collaborative (CPQCC).

I described both the vision and the difficulties I was facing over dinner to friends who were highly successful specialists in organizational development. They responded that what I was trying to develop was a highly complex organization and asked if I had training or experience in this area. I answered that even though my training in Maternal and Child Health had emphasized the importance of putting together multi-stakeholder committees and initiatives, and how to identify stakeholders whose participation would be essential, guidance on how to actually build a complex organization was not part of the curriculum. Their guidance on how to proceed was essential. The first step was to develop the organization’s groundwork: (1) craft an initial mission statement that aligns with each stakeholder’s strategic goals, (2) craft initial key organizational policies that would not only drive the collaborative but would be acceptable to potential partners, and (3) select who to invite as members of the executive committee and issue the invitations. The second step was to conduct face to face meetings with each of the potential executive committee members: (1) rank the potential members with respect to their enthusiasm, and (2) begin one-on-one meetings to present and refine the CPQCC’s mission and organizational policy. In these discussions, we sought to address the question, “what is your strategic mission and how will partnering with CPQCC advance your strategic mission?”—that is, what are the benefits of your organization’s joining the CPQCC? However, it was important to appreciate that while describing all the benefits, the candidate would be thinking about and rarely openly sharing all of the potential downsides. An important but often neglected and absolutely essential part of this discussion is to jointly identify the potential risks that their participation might engender. This will allow understanding of potential risks and begin to construct a risk/benefit “equation” for participation. By understanding their concerns, one will then be in a position to jointly figure out how to maximize benefit and minimize risk. The goal of the face-to-face meeting is to negotiate an agreement with respect to the organization’s name, mission statement, policy, their role as a partner, and most importantly, what specific value their partnership can bring to their organization. In building a complex organization, one needs to move the executive committee from passive stakeholders to active partners. To accomplish this, participation must bring value. A fundamental concept especially germane to a quality improvement collaborative is that value is essential for both the initial behavioral change and maintaining the new behavior. A successful quality improvement organization must be obsessed with continuously identifying ways to provide value for its members and partners. Figure 1 shows our founding partners and the value that their participation would provide.

## 3. Building the Network

### 3.1. Even More Face to Face

After the first round of meeting individually with each potential partner, the next step was to meet with groups of two potential partners who have a history of not working well together. Again, the task was to jointly modify, as needed, the mission statement and organizational policy and to get them to work together to craft how the CPQCC would facilitate reaching their common strategic goal of improving the health of California’s pregnant women and their newborns. We then put together several groups of three potential partners. This was a very time-consuming process. At this point, we were almost a year from the initial invitation to join the executive committee and folks were asking when we were actually going to meet. We had our first meeting a little over a year from issuing the invitations.

Although the initial meetings described above were with state agency and neonatal practice decisionmakers, when we had our first meeting, many of the participants from state agencies were lieutenants rather than leaders with the power to make decisions essential to partnership. In terms of developing a complex organization, this was a huge setback. Fortunately, we were able to identify several powerful advocates who were able to stress the importance of the enterprise and get the agency decision makers to attend.

### 3.2. Building the California Database

An important strategy in building a complex quality improvement organization is to solicit participation from the membership and to assure that their participation results in timely action. We recognized that there were many California neonatologists who were interested in data and its analysis. One of our first quality improvement committees was our database advisory group. We carefully recruited so as to have geographic (north, central, and southern California) as well as academic and private practice representation. Beginning with the VON data format, their first role was to identify what we wanted to know and what constituted the minimal data we needed to gain this knowledge. An additional concern was how to ensure that our data were accurate. To this end, we adopted three strategies. The first was the traditional approach of building range and logic checks into the database. Our second approach was to develop an executive committee of data entry personnel. In California, data entry personnel ranged from professional data entry personnel to neonatologists and NICU nurses. In addition, California NICUs varied widely with respect to census and resource. To assure that we had the needed depth of frontline expertise required having broad-based representation on our committee. Their role was to assure that the data element definitions and data entry procedures were clear, that our entry formats were optimally sequenced and presented so as to minimize data entry errors, and to assess the feasibility of extracting and unambiguously defining suggested new data elements. This committee remains a mainstay even to this day. All database and data collection changes must be approved by the committee. Our third approach to assuring high-quality data is to hold yearly data trainings at several locations throughout California. An essential part of these meetings is to identify problems that members are experiencing in data entry and data closeout as well as to present any new data elements that are scheduled for inclusion. Beginning in 2006, each year, several hundred data entry folks attend these meetings. In addition, we made our database team available on a daily basis to answer data entry questions as well as to reach out to centers that had a high number of missing data items.

### 3.3. The Quest for Value

A successful complex organization must constantly search for ways to increase the value of participation for its members and partners. An early example of identifying ways to provide value was seen with our executive committee whose state participants were in leadership positions within their organizations. We noticed that during the lunch break and at even at the end of the formal executive committee meeting, there would be private conversations among them. Clearly, they were using the meeting for backchanneling. To build value, we stopped the meeting at 2 h rather than the traditional 3.5 h, giving the participants time to do their private business. The result was that we had exceptional attendance, not only to do the work of creating the CPQCC, but also for them to take advantage of the setting for backchanneling.

### 3.4. CPQCC Database Development Proceeded in Several Stages

Phase 1 (VON in California): At its initiation, we used the standard VON designed paper forms, and scanned them into a data file that we cleaned and submitted to the VON. The VON then provided the CPQCC with a custom yearly standard aggregated VON report for California members and provided the members with yearly individual standard VON paper-based performance reports. This had several limitations. Scanning the paper forms into digital format, running error detection, and then having to have the NICUs make corrections to be re-entered was very labor intensive. Our members also wanted to expand the database.

**Figure 2:**
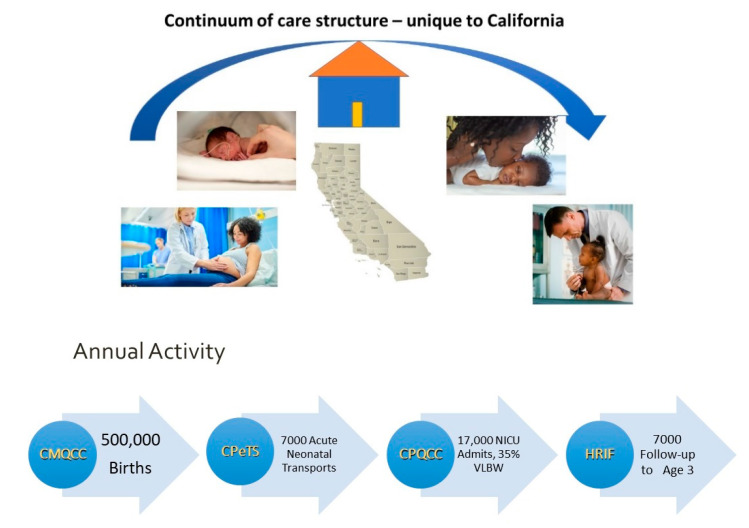
Data to support perinatal quality improvement in California from the CPQCC and California Maternal Quality Care Collaborative (CMQCC) data centers. Images in this figure are licensed for use from iStock.com. NICU: neonatal intensive care unit; VLBW: very low birth weight.

Phase 2 (Addressing California’s needs): The data committee felt that the VON database was a good foundation. They felt the need to include more information on some of the items and to include infants readmitted to the NICU as well as high-risk infants that had birth weights greater than 1500 g. The latter was felt to be essential because in most NICUs, these infants make up the majority of the infants cared for. They also wanted more timely reports and to be able to compare their NICU not only with all California NICUs, but also with California NICUs with the same level of care designation. When we considered all of these new requirements, our systems developer, Dr. Beate Danielson at Health Information Solutions, realized that doing this would require online data submission and real-time report generation. In 2000, we began to process our paper data entry forms, report our expanded database to our members, and submit the “small babies” (birth weight less than 1500 g) subset to the VON. Because the CPQCC is a regional member of the VON, our CPQCC members are also full members of the VON and receive data reports from the CPQCC, enabling comparison with California benchmarks, as well as from the VON, enabling comparison with national benchmarks. This dual source of benchmarking provided additional value to our members. By 2006, our data entry was completely online, and our online data reports were in real time. We also began to compile the yearly mandated CCS NICU activity and outcomes report. Following each NICU’s review and at their request, we submitted the report to CCS. To gain a more complete picture of perinatal risks and outcomes, in 2007, we began to collect and include information and a quality assessment on the approximately 7000 acute neonatal transports [5]. In 2009, we worked with CCS to develop and link NICU data to an all California database that assessed the completeness of NICU registration as well as the social/medical needs and developmental outcomes of NICU graduates until age three in California’s statewide High-Risk Infant Follow-Up program [6,7]. Because the CPQCC had narrowed its focus to NICU care, there was a need to address California’s maternity care. Our sister collaborative, the California Maternal Quality Care Collaborative (CMQCC), was established in 2006 to assess the outcomes of all of California’s 500,000+ births using near-real-time administrative data to facilitate rapid cycle quality improvement [8]. This maternal data was then linked to our NICU data. Figure 2 shows the yearly data that are available to support perinatal quality improvement in California.

**Figure 3:**
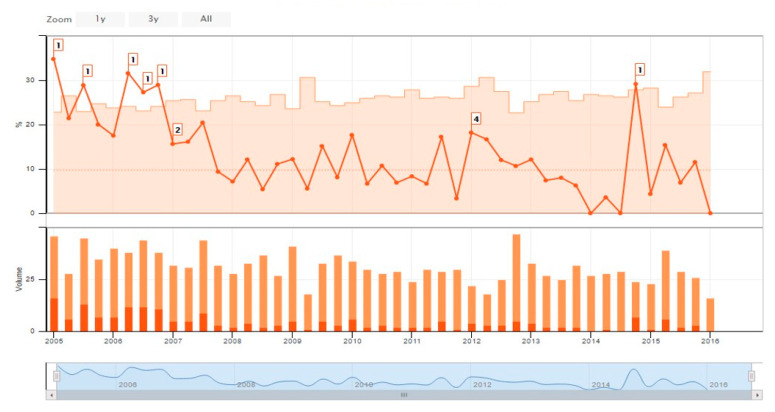
Sample control chart feature for nosocomial infection of a member hospital.

Phase 3 (Enhancing support for our quality improvement activities). Table 1 shows the development of our data system. As we grappled with how to obtain an overall picture of a NICU’s quality, we incorporated the Baby-MONITOR developed by Dr Jochen Profit [9]. In order to support our rapid cycle quality improvement activity, we incorporated control charts as a standard element of our real time online report (Figure 3).

**Figure 4:**
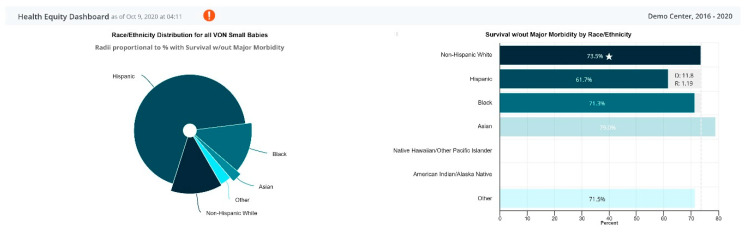
Sample disparity dashboard report.

As we continued to focus on and emphasize our work on health equity, we created a disparity dashboard (Figure 4). We plan to assess how our current efforts in incorporating equity goals into quality improvement may lead to reduced disparities. In addition to the quantitative approach outlined above, over the last several years, we realized that although we could identify NICUs that were challenged or highly successful, identifying the factors that held them back or allowed them to succeed required a qualitative approach. We believe that our developing mixed methods approach can greatly accelerate quality improvement interventions by informing both the where there is need (quantitative) as well as the factors that are important drivers of the need (qualitative) [10,11,12,13,14].

## 4. Turning Data into Action

Three elements are essential to any quality improvement enterprise: Collecting high-quality reliable data, presenting real-time risk-adjusted reports that effectively inform opportunities for quality improvement, and most importantly, providing support to perinatal providers in their work of improving perinatal care and outcomes. Our quality improvement arm of the CPQCC was initiated by Dr David Wirtschafter at the inception of the CPQCC in 1997. A key strategy was to create a Perinatal Quality Improvement Panel (PQIP) to design and manage continuous quality improvement (CQI) cycles. Members were chosen to ensure representation from neonatologists (eleven), perinatologists (four), nursing clinical experts (four), administrators of the Regional Perinatal Programs of California (six), and a health plan physician with expertise in quality improvement (QI). Appointments to the PQIP were designed to balance both academic and private practice constituencies as well as to ensure regional representation. The PQIP guides all of the CPQCC’s quality improvement activities including choosing collaborative topics, creating toolkits, and designing innovative QI models. The PQIP’s subcommittees serve as action arms for the panel by creating and disseminating tools and information that help CPQCC members implement the PQIP’s QI recommendations. From its modest beginning, the PQIP has grown to include four committees on Analysis (to identify quality improvement needs), Education, Research (analyze and publish results from our QI collaborative projects), and QI Infrastructure. These committees meet monthly and are made up of physicians, nurses, and other NICU personnel who, together, contribute more than 100 hours a month as volunteers. Each committee develops a yearly strategic plan and timetable of accomplishments and their status in meeting these goals is presented at each of the quarterly all PQIP meetings. We believe that strategically and when appropriate, rapidly moving from plan to action has been a key to success.

The PQIP began to develop and release quality improvement toolkits that were freely available on the CPQCC’s website [15] from its inception with a commitment to review and update regularly. Our first toolkit, which concerned administration of antenatal steroids prior to preterm delivery, was developed and released within a year of the CPQCC’s inception. Believing in the essential importance of rapidly moving from plan to action, we launched our first full-scale quality improvement initiative on improving the use of antenatal steroids in 1999 [16]. The format of our approach to formal statewide QI initiatives has also evolved over time. Our highly successful initial approach developed by Dr. Wirtschafter was based on identifying topics with improvement potential, assessing the validity of potential improvement strategies, and then developing readily available web-based toolkits and launching statewide educational lectures, webcasts, and workshops across California and at the CAN annual meeting. These sessions included small group discussions addressing implementation goals, barriers faced, and ways to overcome them. NICUs registered to participate. However, the actual implementation was conducted individually at each NICU without formal oversight. Although the toolkit/workshop approach was highly successful [17], CPQCC shifted to the Institute for Healthcare Improvement (IHI) Model [18].

The goals of adapting the IHI model were to build practical improvement capacity based on the science of improvement into every CPQCC NICU, healthcare executive, and clinician, while driving innovation to dramatically improve performance at all levels of the health care system. The advantages of the IHI model include commitment to a specific level of improvement within a specified timeframe; accelerating achievement from a community of learning approach; the use of Plan Do Study Act (PDSA); cycles of intervention monitored by recording agreed upon process, outcome, and balancing measures on a run chart; and each NICU’s reporting of their progress to the group on monthly meetings. Movement to several elements of this approach was successfully demonstrated by Dr. Wirtschafter with the statewide CCS/California Children’s Hospital/CPQCC catheter-related infection initiative. As the final report points out, it was clear that the participants that routinely conducted multiple audits during the course of their projects (a precursor to the IHI PDSA-run chart approach) were the most successful. To fully integrate the IHI approach into the CPQCC, the leadership of CPQCC’s quality improvement arm transferred to Dr. Paul Sharek in 2008. Although not a neonatologist, Dr. Sharek was highly experienced with the IHI breakthrough approach. He also introduced the use of quality improvement approaches to the work conducted by the PQIP committees. A difficulty with many committees is the disconnect between discussion and action. By employing yearly strategic goals, a timetable for their accomplishment, and monthly reporting of progress, members of our committees experience their discussions and plans actually leading to timely action. This approach is not only important to the membership who will benefit from the action but is highly motivational to the members who volunteered to participate in the committees. As shown in Table 2, the CPQCC has conducted six highly successful QI Collaborative based on the IHI model over the past 10 years. The first collaborative using this approach, Healthcare-Associated Infections was from February 2008 to January 2009. The 19 NICUs in the Healthcare-Associated Infections Collaborative decreased catheter-associated bloodstream infections (CABSIs) by 75% in infants with birth weights ≤ 1500 g.

In October 2018, leadership was transferred to Courtney Breault, a neonatal nurse with expertise in quality improvement who had served as codirector of the Quality arm since its inception, and Dr. Jochen Profit, a neonatologist and health services researcher expert in quality improvement. Several challenges that they faced were how to effectively reach out to vulnerable NICUs who had not participated or rarely participated in our formal QI programs, how to address racial and socioeconomic equity into our quality improvement work, and how to meet the data and quality improvement needs unique to certain CPQCC NICUS. These include addressing the specific needs of NICUs of our high-volume children’s hospitals with their many surgical cases and also addressing the needs specific to our small low-volume NICUs. To identify their specific needs, the PQIP recommended that two workgroups be created. Based on their feedback and input, the CPQCC has created QI initiatives tailored to their needs.

In order to better meet the quality improvement needs of our diverse NICUs, we have moved away from the biannual all-NICU formal initiative approach of the past to several concurrent initiatives specifically designed to meet the diverse needs of our member NICUs. In 2021, in addition to an all-newborn ICU offering neuroprotective care, we will have two growth and nutrition initiatives, one designed to meet the needs of smaller low-volume NICUs, and a second to meet the needs of postoperative surgical infants cared for in our Children’s Hospital NICUs. In addition, we are incorporating the use of vignettes to identify the extent and areas of decision-making variability. This approach, which has been led by Dr. Kurlen Payton, allows our QI facilitators to identify and work on those areas of decision-making that require greatest attention. A further advance in our approach to QI is the incorporation of the ECHO model that will be piloted by a team that includes Dr. Payton and Dr. Henry Lee in our upcoming Optimizing Antibiotic Stewardship Initiative.

## 5. Quality Improvement Research and Training

It is important to note that the principle focus of a quality improvement collaborative and its database should always be quality improvement. As many CPQCC members are academic centers and all neonatologists receive research training during fellowship, research using the infrastructure of the CPQCC has always been a consideration. However, as the primary mission of the CPQCC is quality improvement, the efforts of research need to be considered an important but secondary goal. In that context, we also recognize that as a large statewide QI organization, there are opportunities to advance science in several ways. First, we have aimed to lead the efforts of QI science. It is important to evaluate the effectiveness of QI interventions and learn from past projects in order to inform future QI work. We consider it an obligation to formally study QI in order to benefit both CPQCC members and the larger QI community. Second, the infrastructure of data collection in the CPQCC allows for opportunities in both observational and prospective clinical research. These have included supplemental data collections, epidemiologic studies, health services research, and the facilitation of clinical trials.

## 6. Conclusions

We end with some of the lessons learned from the development of the CPQCC:

1. Perinatal Quality Collaboratives (PQCs) are complex organizations that rely on the effective contribution of partners who may have differing agendas and approaches to the common goals of the collaborative. Engaging an expert in organizational development can greatly facilitate developing a coherent, productive collaborative.
2. Identifying and increasing value is the essential driver of participation, behavioral change, and maintaining the new behaviors. An ongoing task for successful PQCs is to continuously look for ways to increase value for members and stakeholders.
3. Rapidly moving from committee discussion to action creates dynamic energy for the membership as well as a sense of accomplishment for the members of the committees. Avoid spending lots of time to plan the perfect intervention. The IHI approach of multiple tests of change will refine the project as it proceeds by incorporating approaches that work and rapidly abandoning approaches that do not.
4. Using the smart aim approach [22] to define specific goals within a specified timeframe, and formally reviewing progress on a quarterly basis, at a minimum, is an essential strategy across the enterprise. This is especially important for volunteer committees as it facilitates reaching their defined objectives within a reasonable timeframe. This provides not only a useful product for the members but also instills a sense of motivating accomplishment to the volunteers.
5. Seek the expertise needed for success in your front line. Whereas the leadership can set out the destination, it is the frontline folks who have the working experience to understand what has to be changed and how it might most efficiently be changed to reach the destination.
6. When designing a quality improvement initiative, make sure that all of the elements required by the SQUIRE publication criteria will be met. A critique of this sort will often strengthen final intervention design, data collection, and analytic strategy.

## Figures and Tables

**Table 1:** CPQCC Database Development.

Year	Data/Feature
1998	VON < 1500 g
2000	High-risk > 1500 g
2007	Real time reporting + neonatal transport
2008	Infants linked across NICUs
2009	Statewide high-risk follow-up until age 3
2013	NICU based follow-up records
2017	Real-time control charts
2019	Baby-MONITOR
2019	Health equity dashboard

VON: Vermont Oxford Network.

**Table 2:** CPQCC Quality Improvement (QI) collaborative projects based on the Institute for Healthcare Improvement (IHI) Model for Improvement.

Project	Dates	Number of NICUs	Summary	Result
Healthcare-Associated Infections	Feb 2008–Jan 2009	19	Aim to decrease catheter-associated bloodstream infections in very low birth weight infants	Reduction of 75% in infection rates
Breastmilk Nutrition Collaborative	Sep 2009–Apr 2011	11	Increase breastmilk feeding rates for very low birth weight infants	Participants increased breastmilk feeding rates at discharge to home from 54.6% to 64% and decreased necrotizing enterocolitis rates from 7% to 2.4% [19]
Delivery room management	Jun 2011–Nov 2012	20	Improve management for high-risk deliveries with focus on thermal management, reducing invasive ventilation, and supporting teamwork	Collective decrease in admission hypothermia, delivery room intubation [20]
Optimizing length of separation	Jun 2013–May 2015	20	Reduce length of stay for infants born between 27–32 weeks gestational age	Participants decreased length of separation by average of 3 days and increased early discharge (before 36 weeks, 5 days) from 41.9% to 31.6% [21]
Antibiotic stewardship	Jun 2016–Nov 2017	28	Reduce antibiotic utilization rates through bundle including antibiotic “time-outs”	Estimated to have reduced antibiotic days by 11,700 and decreased antibiotic utilization rate by 13.8%
